# High prevalence of human papillomavirus infection in HIV-infected women living in French Antilles and French Guiana

**DOI:** 10.1371/journal.pone.0221334

**Published:** 2019-09-04

**Authors:** Sylvie Abel, Fatiha Najioullah, Jean-Luc Voluménie, Laetitia Accrombessi, Gabriel Carles, Dominique Catherine, Déborah Chiappetta, Cyril Clavel, Akua Codjo-Sodokine, Myriam El Guedj, Janick Jean-Marie, Vincent Molinié, Sandrine Pierre-François, Sofia Stegmann-Planchard, Vincent Vantilcke, Tania Vaz, Mathieu Nacher, André Cabié, Raymond Césaire, for HP2V study group

**Affiliations:** 1 Infectious Diseases Department, CHU de Martinique, Fort-de-France, France; 2 Université des Antilles, Fort-de-France, France; 3 Virology Laboratory, CHU de Martinique, Fort-de-France, France; 4 Gynecology Unit, CHU de Martinique, Fort-de-France, France; 5 Obstetrics and Gynecology Unit, Centre Hospitalier "Andrée Rosemon", Cayenne, France; 6 Obstetrics and Gynecology Unit, Centre Hospitalier de l’Ouest Guyanais Franck Joly, Saint-Laurent-du-Maroni, France; 7 Pathology Department, CHU de Martinique, Fort-de-France, France; 8 Inserm CIC1424, CHU de Martinique, Fort-de-France, France; 9 Infectious Diseases Unit, Centre Hospitalier Louis Constant Fleming, Saint-Martin, France; 10 Gynecology Unit, Centre Hospitalier Louis Constant Fleming, Saint-Martin, France; 11 Day Hospital Unit, Centre Hospitalier « Andrée Rosemon », Cayenne, France; 12 Infectious Diseases Unit, Centre Hospitalier de l’Ouest Guyanais Franck Joly, Saint-Laurent-du-Maroni, France; 13 Inserm CIC1424, Centre Hospitalier « Andrée Rosemon », Cayenne, France; Katholieke Universiteit Leuven Rega Institute for Medical Research, BELGIUM

## Abstract

An association between HIV infection and cervical cancer, a major public health issue worldwide, has been reported. The aim of this study was to estimate the prevalence of human papillomavirus (HPV) infection and the distribution of HPV genotypes in HIV-infected women living in French Antilles and Guiana and to determine HIV-related characteristics associated with HPV infection. This cross-sectional study included 439 HIV-infected women who were followed between January 2011 and May 2014. Variables related to HIV infections were collected, and cervical samples were analysed to determine HPV genotypes. The median age of the population was 46 years. Estimated prevalence of HPV and high-risk (HR)-HPV infection were 50.1% IC95 [45.4–54.7] and 42% IC95 [37.3–46.6], respectively. HR-HPV 16, 52, 53 or intermediate risk-HPV-68 were found in 25% to 30% of the HPV-infected patients. Gynaecological screening revealed abnormal cervical smear in 24% and 42% of HR-HPV-negative and HPV-positive women, respectively (p = 0.003). Approximately 90% of women were on antiretroviral therapy (ART). Demographic characteristics associated with a higher prevalence of HPV infection included alcohol consumption. Regarding HIV-related characteristics, current therapy on ART, its duration, and undetectable plasma concentrations of RNA-HIV1 were associated with a lower risk of HPV infection. Infection rate with HR-HPV was higher than what is commonly reported in HIV-negative women worldwide and was more likely in women with incomplete HIV suppression. These results highlight the need for supporting adherence to ART, cervical cytology, HPV testing and HPV vaccination.

## Introduction

Cervical cancer is a major health issue worldwide. In 2012, 528,000 new cases were estimated and approximately 270,000 women died from cervical cancer [1]. Approximately 85% of these deaths occurred in low- and middle-income countries where screening is not available or not practised routinely and where the access to health services is limited. Human papillomavirus (HPV), a highly prevalent sexually transmitted virus, has been linked to several cancers, such as vaginal, vulva, head and neck, anal, and penile carcinomas [2], and has been recognized as the aetiological agent of cervical cancer [3]. Most HPV infections are transient due to spontaneous virus clearance within a few months, with results depending on geographical regions where the studies took place, most likely due to methodological differences including sampling or HPV detection [4]; this phenomenon is also associated with increasing age [5]. The risk of cervical cancer is strongly associated with the persistence of HPV (>12 months) and high-grade squamous intraepithelial lesions (HSIL) [6].

HPV genotypes have been classified according to their oncogenic potential [7]. In the high-risk (HR) group HPV-16, 18, 31, 33, 35, 39, 45, 51, 52, 56, 58, 59 are classified as carcinogenic (group 1) while HPV 68 is classified as probable carcinogenic and HPV-26, 53, 66, 67, 70, 73, and 82 as possibly carcinogenic (group 2 or intermediate risk) [7]. HPV-6, 11, 40, 42, 43, 44, 54, 61, 72 and 81 belong to the low-risk (LR) group (group 3) [7]. HPV-16 and 18 account for 70% of all invasive cervical cancers worldwide [8]. Given the major role of HPV in the risk of cervical cancer, bivalent (HPV-16, 18) and quadrivalent (HPV-6, 11, 16, 18) vaccines have been developed and recommended. A nonavalent vaccine (quadrivalent vaccine supplemented with HPV-31, 33, 45, 52 and 58) has been approved in 2014 by the American Food and Drug Administration and is now recommended by the European Medicines Agency (EMEA).

The overall prevalence of HPV infection varies greatly among populations, with a worldwide heterogeneity dependent on behaviours and economic levels [8]. Because HPV is sexually transmitted, the impact of and on other sexually transmitted infections (STIs), such as human immunodeficiency virus (HIV), is a major health issue. A recent meta-analytic review based on 14 studies indicates that the risk for HIV acquisition is doubled in HPV-infected women, regardless of the oncogenic potential of HPV (HR or LR), and that HIV acquisition increased with the number of HPV types [9]. These observations are in agreement with the expected impact of HPV on cell adhesion and infection-induced inflammation in the genital tract [10]. However, such analyses may be confounded by sexual activities and lifestyle, and a nested case-control study including East African women did not find any association between HIV acquisition and HPV-infection [11]. On the other hand, studies performed in women screened for cervical cancer in Southern Africa showed that detection of HPV infection increased rapidly within the first years after HIV seroconversion [12] and that being HIV-positive was associated with a 4-fold increased risk for HR-HPV infection [13], thus supporting a possible reverse causality. Whatever the respective roles of HPV and HIV in promoting co-infection, it has been shown that HPV persistence and the subsequent carcinogenic risk depends on the host immune response [14], and an association between HIV/AIDS and cervical cancer has been reported in Europe and the United States [15,16] and, although less documented, in Africa [17]. Overall, the data mentioned above support the need for better defining the prevalence and characterisation of HPV infection in HIV-infected populations, and the role of the patients’ HIV status.

The aim of this study was to estimate the prevalence of HPV infection and the distribution of HPV genotypes in HIV-infected women in French Antilles and French Guiana where HPV infection is highly prevalent compared with other countries worldwide [18] or with metropolitan France [19] where healthcare system is similar. HIV-related characteristics associated with a high risk of HPV infection were also studied.

## Patients and methods

This was a cross-sectional study of the HPV genotype’s prevalence in a collection of biological samples and its associated de-identified data. Women were followed between January 3^rd^ 2011 and May 2^nd^ 2014 in four hospitals in the French Antilles (University hospital of Martinique, Hospital of Saint-Martin) and French Guiana (Hospital of Cayenne, Hospital of Saint-Laurent du Maroni) at the time of their annual visits scheduled for gynaecological follow-up. All patients included in the study gave informed consent for HPV testing and cervical-uterine smear. Because screening of the patients was made in the context of their routine follow up for HIV infection, oral non-opposition was considered for sample collection. Written consent was given for the inclusion of data in the French national HIV Nadis database. The study was approved by the Comité de Protection des Personnes (CPP) Sud-Ouest et Outre Mer III ethics committee. ClinicalTrials.gov identifier NCT01226368.

### Study population

Patients’ inclusion criteria were to be at least 18 years old, to be followed for HIV1 or HIV2 infection in one of the participating centres and to have an indication for cervical-uterine smear analysis for routine screening in the context of HIV infection follow-up, or any cervical-uterine pathology. Exclusion criteria included a history of total hysterectomy and the impossibility of obtaining smear samples.

### Variables assessed and cervical analysis

Variables recorded were demographic characteristics, alcohol and drug consumption, history of HIV infection (time since diagnosis, mode of HIV acquisition) and medical history (co-infections, gynaecological diseases or surgery). Regarding HIV infection, CD4 counts at the beginning of the study, CD4 nadir, HIV1-RNA load and status regarding past and current antiretroviral therapy, if any, were collected. Macroscopic examination of the cervix with acetic acid or lugol’s iodine was reported.

Cervical samples were taken using the ThinPrep-PreservCyt solution kits (Hologic). Samples were then sent to the pathology laboratory of Martinique University hospital for a centralized assessment and, after processing for cytological study (Hologic), to the virology laboratory. The detection and genotyping of HPV-DNA was performed using the Papillocheck kit (Greiner-Bio-one) after DNA extraction on EasyMag (Biomérieux). This kit allows the identification of HPV genotypes including HR or group 1 (16, 18, 31, 33, 35, 39, 45, 51, 52, 56, 58, 59) intermediate risk or group 2 (53, 66, 68, 70, 73, 82) and LR or group 3 (6, 11, 40, 42, 43, 44, 55) genotypes. Abnormal cervical smear analysis identified atypical squamous cells of unknown significance (ASCUS), low-grade (LSIL) or high-grade (HSIL) squamous intraepithelial lesions according to the 2014 Bethesda classification [20].

### Primary and secondary objectives

The primary objective of the study was to estimate the prevalence and the distribution of HPV genotypes in the cervical smear of women infected with HIV. Secondary objectives included specification of the risk factors associated with HR HPV infection among HIV-infected women, and the relation with parameters associated with HIV control (CD4 level, viral load).

### Statistical analysis

Analysis was performed using Stata 12 software (StataCorp LP, College Station, TX, USA). Categorical variables were summarized using frequencies and percentages and compared using Fisher Exact test. Continuous variables were summarized using medians and interquartile ranges (IQR) and compared using non-parametric tests (Mann-Whitney or Kruskal-Wallis test, when appropriate). The association between HPV infection and genotypes, and demographic- or HIV-related variables was assessed using the two-sample Wilcoxon rank-sum test. Logistic regression was used to identify factors associated with group 1 HPV infection and to estimate odds ratios (ORs) and 95% confidence intervals (CIs) for the associations between baseline variables and group 1 HPV infection. Variables with p values <0.20 were introduced in the multivariate logistic regression model. A manual backward stepwise approach was used to remove non-significant variables; only variables with p values <0.05 were retained in the final model. Interactions were sought by introducing interaction terms in the logistic regression model and testing for their significance at the 0.05 level.

## Results

### Cervical analysis, HPV status and HIV-related characteristics of the whole study population

**Fig 1:**
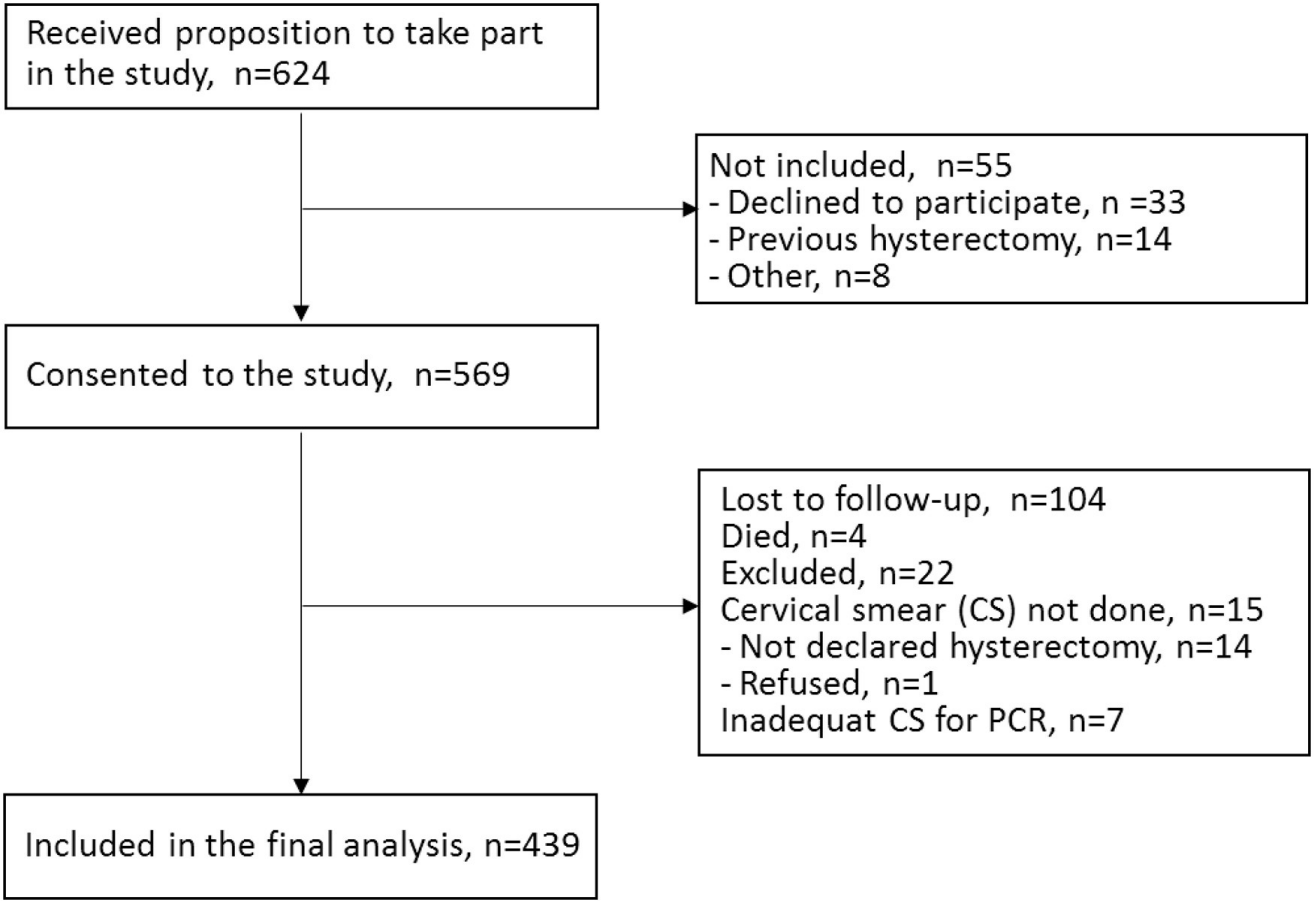
Enrolment and follow-up of the study participants.

Based on the inclusion and exclusion criteria, 569 patients were enrolled. After exclusion of patients with unavailable or inadequate cervical smears and those deceased or lost to follow-up, 439 women (197 in Martinique, 140 in Saint-Martin, 102 in French Guiana) were included in the analysis (Fig 1). Women excluded were not significantly different in terms of HIV status (CD4 counts, time since HIV infection diagnosis and duration of antiretroviral therapy [ART]). The median age at the start of the study was 46 years (IQR 36–53), and the median time since HIV infection was 10 years (IQR 5–16) (Table 1).

**Table 1:** Demographics and HIV-related parameters in the population according to HR-HPV status.

Parameters	Total (n = 439)	HR HPV positive (n = 140)	HR HPV negative (n = 299)	Unadjusted Odd ratio [95% CI]	p
Median age [IQR], yrs	46 [38–53]	45 [35–53]	46 [39–53]	0.99 [0.97–1.01]	0.2
Median HIV duration [IQR], yrs	10 [5–16]	9 [4.5–16]	11 [6–16]	0.98 [0.95–1.01]	0.1
AIDS, n (%)	109 (24.8)	40 (28.6)	69 (23.1)	1.33 [0.85–2.10]	0.2
Current ART					
Current ART, n (%)	380 (86.6)	114 (81.4)	266 (89.0)	0.54[0.31–0.95]	0.036
Median ART duration [IQR], months	21 [9.5–43]	16.5 [5–35]	23.5 [11–45]	0.98 [0.97–0.99]	0.004
CD4 T cells count					
Median [IQR], cells/mm^3^	530 [418–691]	509 [352–683]	539 [429–700]	0.99 [0.99–1.00]	0.06
< 200 cells/mm^3^, n (%)	25 (5.7)	12 (8.6)	13 (4.4)	2.06 [0.92–4.64]	0.08
< 500 cells/mm^3^, n (%)	182 (41.5)	63 (45.0)	119 (39.8)		0.1
Median [IQR] nadir, cells/mm^3^	185 [56–300]	182 [54–322]	186 [57–290]	1.24 [0.82–1.86]	0.7
HIV RNA					
Median [IQR], log copies/ml	1.60 [1.30–1.75]	1.60 [1.30–2.39]	1.57 [1.30–1.60]		0.03
<50 copies/mL, n (%)	312 (71.1)	84 (60.0)	228 (76.3)	0.47 [0.30–0.72]	0.001
Other infections, n (%)					
Herpes	31 (7.1)	9 (6.4)	22 (7.4)	0.87 [0.39–1.93]	0.8
Gonorrhea	0	0	0		-
Syphilis	26 (5.9)	9 (6.4)	17 (5.7)	1.14 [0.49–2.62]	0.8
Abnormal pap smear before study, n (%)*	86 (28.8)	35 (41.7)	51 (23.7)	2.30 [1.34–3.92]	0.003
Consumptions, n (%)					
Tobacco	77 (23.5)	33 (29.7)	44 (20.3)	1.66 [0.98–2.81]	0.07
Alcohol	49 (15.1)	23 (20.7)	26 (12.2)	1.88 [1.01–3.87]	0.05
Psychoactive drugs	28 (8.7)	12 (10.9)	16 (7.6)	1.50 [0.68–3.29]	0.3
Contraception, n (%)	140 (46.4)	36 (35.6)	104 (51.7)	0.52 [0.32–0.85]	0.01
Condom, n (%)					
Always	118 (37.8)	30 (28.3)	88 (42.7)	0.53 [0.32–0.88]	0.01
Sometimes	7 (2.2)	2 (1.9)	5 (2.4)	0.77 [0.15–4.05]	1
Never	187 (59.9)	74 (69.8)	113 (54.9)	1.90 [1.15–3.13]	0.01
Combined oral contraceptives, n (%)	17 (5.4)	5 (4.7)	12 (5.8)	0.81 [0.28–2.36]	0.8
Intrauterine devices, n (%)	12 (2.7)	3 (2.1)	9 (3.0)	0.71 [0.19–2.65]	0.8
Educational level, n (%)					
High	37 (10.6)	9 (8.2)	28 (11.7)	0.68 [0.31–1.50]	0.4
Secondary	106 (30.4)	36 (33.0)	70 (29.2)	1.20 [0.74–1.95]	0.5
Primary	206 (59.0)	64 (58.7)	142 (59.2)	0.98 [0.62–1.55]	1

HR HPV: high-risk human papillomavirus types

*Data available for 299 (total), 84 (HPV group 1 positive) and 215 (HPV group 1 negative) women.

Approximately 90% of patients were on ART, and HIV RNA load was undetectable in plasma (<50 copies/ml) in 71% of patients. CD4 counts were greater than 500/mm^3^ in 55% of patients with a median nadir CD4 of 185 cells/mm^3^ (IQR 56–300). The consumption of alcohol or drugs and smoking were reported in a minority of patients. Macroscopic examination and cytological analysis performed in the context of gynaecological visits revealed abnormal cervix and abnormal smears in 24% and 34% of patients, respectively. Abnormal smears mainly included ASCUS or LSIL and, to a lesser extent, HSIL (Table 2).

**Table 2:** Cervical status according to the HR-HPV-infected women at inclusion.

Cervical and HPV status of the population	Total (%) (n = 439)	HR-HPV positive (n = 140)	HR-HPV negative (n = 299)	p
Cervical analysis (gynecological visit*), n (%)				
Abnormal	105 (24.3)	42 (30.4)	63 (21.4)	0.05
**Cervical cytology, n (%)**				
Abnormal	149 (33.9)	80 (57.1)	69 (23.1)	<0.001
ASC-US	49 (11.2)	23 (16.4)	26 (8.7)	0.02
ASC-H	5 (1.1)	5 (3.6)	0	0.003
LSIL	35 (8.0)	28 (20.0)	7 (2.3)	<0.001
HSIL	5 (1.1)	5 (3.6)	0	0.003
Other	56(12.8)	19 (13.6)	37 (12.4)	0.7

HR HPV: high-risk human papillomavirus types

*7 missing data.

Based on their medical history, less than 10% of the population exhibited herpes or syphilis co-infection. Contraception, when applied, mainly consisted in condom use, with very low rates of oral contraceptive use (Table 1).

**Fig 2:**
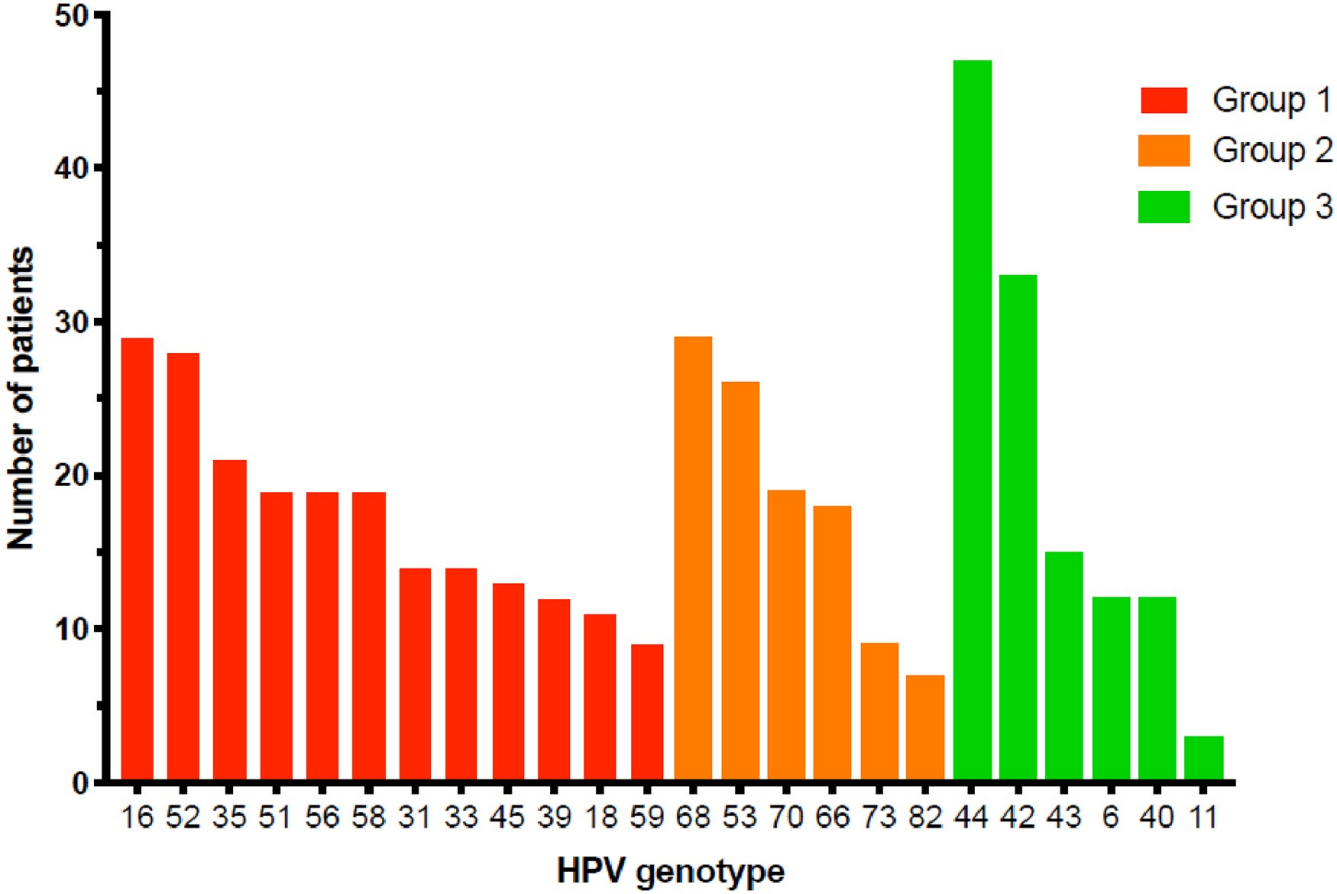
Distribution of HR-HPV (group 1), intermediate risk-HPV (group 2) and LR-HPV (group 3) genotypes.

**Fig 3:**
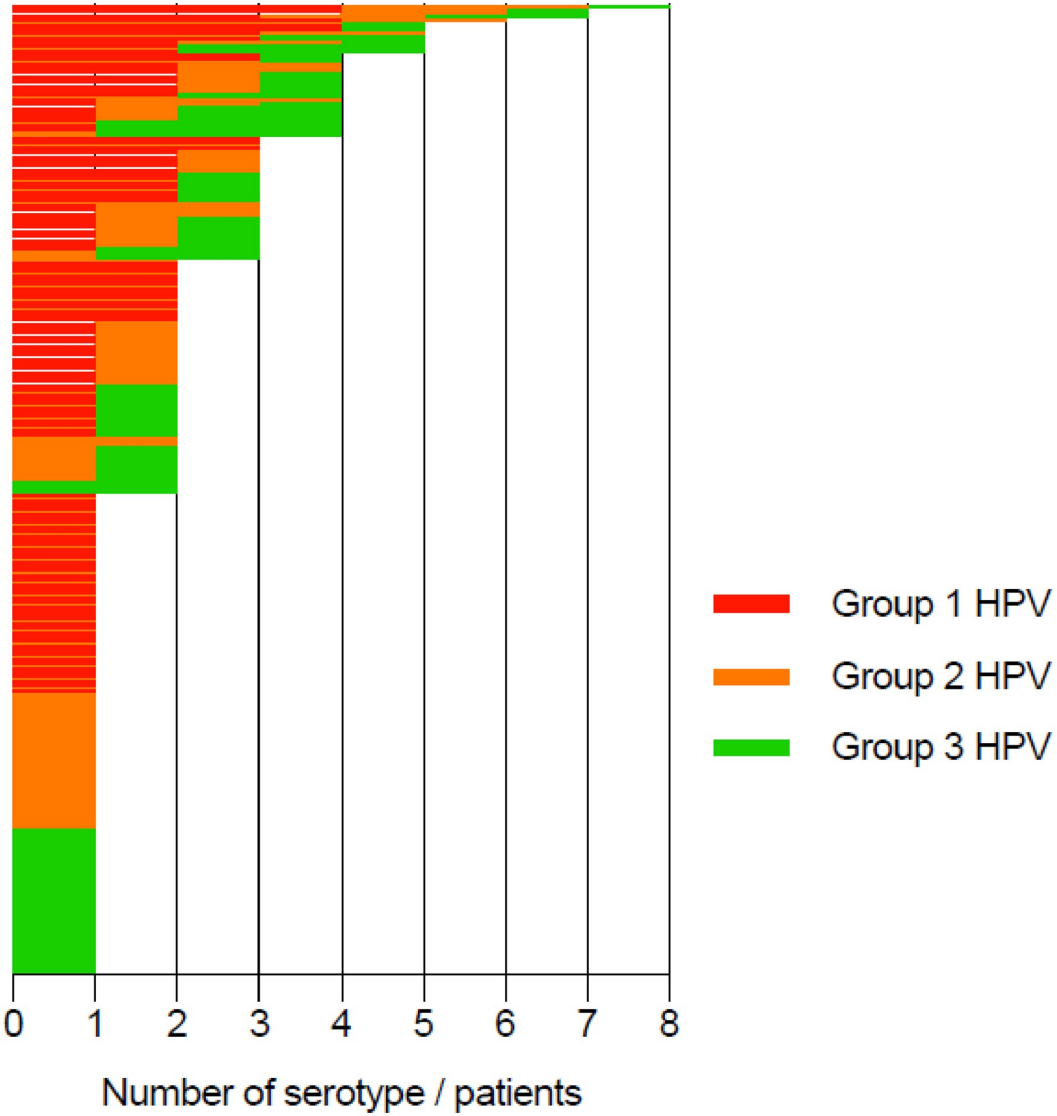
Number of HPV serotypes per HPV infected women according to HPV group.

The estimated prevalence of HPV infection was 50.1% (IC95% [45.4–54.7]), with no significant difference between Antilles and Guiana centres (48.4% and 55.9%, respectively; p = 0.2). The distribution of HPV genotypes revealed that 63.6% of HPV were HR-HPV (estimated prevalence 31.9% IC95% [27.7–36.4]) with the predominance of HPV 16, 52, and intermediate risk-HPV 68 found in 25% to 30% of HPV-infected patients (Fig 2). HPV 18 was found in only 11% of the HPV-infected population. Half of HPV-infected women were infected with several HPV genotypes (Fig 3).

### Characteristics associated with HR-HPV infection

Regarding patients’ demographics and behaviours, alcohol consumption was significantly associated with HPV infection (Table 1). A high educational level was associated with a lower risk of infection, but the difference did not reach the statistical level. Abnormal cervical smear was related to the detection of HR-HPV while an inverse relationship was found regarding condom use, which was confirmed by multivariate analyses (1.93 [1.02–3.66] and 0.44 [0.22–0.88], respectively).

The durations of HIV infection were similar in patients with positive or negative HPV detection, as were the proportions of patients having experienced AIDS (approximately 25%). However, being currently on ART, the duration on ART and undetectable plasma concentrations of HIV1-RNA were significantly associated with a lower risk of HPV infection. There was a negative correlation between low CD4 counts and HR-HPV infection, but that did not reach statistical significance. A significant inverse relationship between mean CD4 counts and HPV was found when considering HPV irrespective of HR or LR type (513 [393–667] vs. 545 [428–728], p = 0,022).

## Discussion

This study reports the prevalence and genotypes of HPV in HIV-infected women living in the French Antilles and Guiana. HPV infection was found in half of the study population, which mainly corresponded to HR-HPV. These results are in agreement with those reporting that HR-HPV was present in about 50% of HIV-infected women living in Tanzania [21] or in Brazil [22,23]. A higher prevalence (78%) of HR-HPV infections has been reported in HIV-infected Black Caribbean women living in the Bahamas [24]. A meta-analysis addressing the prevalence and genotype characterisation of HPV in HIV-infected women in the five continents suggests that the prevalence observed in the present study is close to that found in women with normal cytology living in Africa and Latin America (up to 57%–64%) but higher than what has been reported in women living in Asia, Europe, or North America (25%–34%) [25], supporting geographical behaviour-related specificities, the possible influence of the level of HIV-induced immunodeficiency from a population studied to another, or a different susceptibility to HPV. Indeed, several studies performed in Africa [26], Asia [27], and Latin America [28] have shown different susceptibilities to HPV and risks of HPV-induced cancers dependent on the ethnic origin and the expression of HLA-DRB1/DQB1 alleles. Such an analysis was not included in the design of the present study.

The estimated rate of HPV infection was also higher than that reported in HIV-negative women living in the same area. A study conducted in the French Caribbean island of Guadeloupe showed a prevalence of HR-HPV of 36% among healthy women [29], and two recent studies reported that HPV prevalence in French Guiana reached 35% in women with normal cytology [30,31]. Based on this indirect comparison between HIV-positive and HIV-negative women, and despite the fact that the expected high rate of HPV infection in the French Caribbean population [29] could have minimized the impact of the co-infection, the present study strengthens the concept of an association between HIV and HPV infections. A study carried out in HIV-positive and HIV-negative women living in Benin found a lower prevalence of HPV infection than that reported in both the present and the Tanzanian studies, which, however, remained higher than in the comparative HIV-negative group of the study (31% vs. 23%) [32]. This observation is presumably due to different status of the population assessed regarding characteristics associated with increased risks of HPV infection, which was not the objective of the Beninese study. Overall, these data support an increase in HPV acquisition in HIV-infected women.

In the present study, one third of cervical smears showed abnormal cytology, which is far greater than in HIV-negative women in French Guiana where the proportion is 10% and is already considered high [33]. For HSIL, the proportion was the same as that reported in HIV-negative French Guianese, which is 4 times greater than what was described in HIV-negative women living in metropolitan France [34]. In the present study, HPV mainly corresponded to HR-HPV. The high proportion observed, compared to that reported in HIV-negative populations living in the same [29] or a different area [21,25], is consistent with the increased risk of HIV-infected individuals for developing several cancers and HPV-related pre-malignant intraepithelial neoplasia [35–38]. While a high HLA-related susceptibility to HPV may not be excluded [27,28], it could not account for the difference observed between the two groups of the same area.

A meta-analysis based on 21 studies including HIV-infected African women with invasive cervical cancer (ICC) showed that HPV16, found to be highly prevalent in the present study, was the most frequently detected type (42.5%), followed by HPV18, HPV45, and HPV35 [8]. The high prevalence of HR-HPV-52, 53 and 68 also reported in the present study has been observed in HIV-negative French Guianese women with normal cytology [30]. The prevalence of HPV-52 is similar to that reported in Tanzania [21] and in a study performed in Thai HIV-infected women on combination ART [39], but the high prevalence of HPV-68 was not observed in the Tanzanian and Thai cohorts, which reinforces the idea that the distribution of HR-HPV could be dependent on the HIV status and on the geographical origin of the cohorts.

The burden of HPV-related cancers can then be expected to increase in HIV-seropositive patients, and both American and European guidelines recommend pap test for cervical cancer screening for all HIV-positive women older than 21 years of age or within one year after initiation of sexual activity [40,41]. In HIV-infected women up to age 26, HPV vaccination with nona-, quadri- or bivalent vaccine is recommended [40]. Highly prevalent HPV-types revealed in the present studies are not covered by available vaccines, suggesting that a substantial proportion of the women included may have not been adequately protected by vaccination. However, the high prevalence of HR-HPV-52 and, to a lesser extent, of HPV-31 and HPV-33 observed in the present study, coupled with the rates of multiple HPV infections reported in HIV-positive ICC [42], support the use of the nonavalent vaccine recommended by the European AIDS Clinical Society [41]. While the efficacy and the rationale of vaccination are questionable when HPV infection is established [41], it cannot be excluded that women infected by one specific HPV genotype could still benefit from the protection of the vaccine against other genotypes.

The present study did not find any association between HPV positive status and the time since HIV diagnosis. However, HPV infection was statistically associated with the absence of ART and with characteristics such as low CD4 counts or nadir CD4 known to be associated with a lack of ART efficacy or a poor compliance. These data are consistent with those reported in the cross-sectional hospital-based study involving HIV-infected women in Tanzania [21], showing that a significant HPV positivity was associated with low CD4 counts, and a duration of ART use less than 6 months (RR: 1.28 [1.00–1.036]). The study performed in Thailand in a cohort of HIV-infected women showed that younger age, low CD4 cell-counts and low education were independently associated with HR-HPV infection [37]. Taken together, our data (mainly based on HR-HPV infections) and those previously reported in cohorts of various countries in Africa are in agreement with the increased HPV-induced risk of cancers in patients with CD4 < 500/mm^3^ and with a protective effect of ART on HIV-associated malignancies in compliant women [43]. A meta-analysis of 31 studies showed that women living with HIV on ART had a lower prevalence of high-risk HPV and an increased likelihood of SIL or CIN regression than untreated women after adjustment for CD4 counts and ART duration, suggesting that the protective effect of ART implies both early initiation and sustained compliance [44]. One of the studies included in the analysis found that clearance of oncogenic HPV-positive SIL associated with ART was lower in non-adherent women [45]. Taken together, the reports mentioned above and the present study suggest that factors leading to a sub-optimal immune response to ART, regardless of the cause (sub-optimal efficacy of the current regimen, poor compliance), could favour HPV infection and its related complications and that, conversely, early ART initiation and sustained compliance is expected to reduce the progression of SIL and CIN and incidence of invasive cervical cancer. Given the successful prolongation of life due to ART-mediated HIV suppression, the potentially longer duration of HPV persistence, and the accumulation of somatic mutations and epigenetic changes that contribute to carcinogenesis, maintaining virological control and high CD4 levels is a major issue in HPV-infected women.

The main limitation of the study is the lack of a control HIV-negative group, which did not allow a direct assessment of the impact of the HIV infection on the prevalence and characterisation of HPV infection. It cannot be excluded that the ethnicity of populations was different dependent on the HIV status and that the higher prevalence of HPV infection in HIV-infected women may be due to a greater genetic susceptibility. However, while an ethnic influence may be addressed, the interpretation is expected to be poorly biased when considering women living in the same area. Finally, one may not exclude that visual inspection or cervical cytology may be result in cervical disease misclassification. However, cervical samples have been taken by gynaecologists and have been sent to one pathology laboratory for a centralized assessment, which limit biases on interpretation.

In summary, this prospective cross-sectional study shows that in the population of the French Antilles and Guiana, half of the HIV-infected women are co-infected with HR-HPV, which corresponds to a higher prevalence than that reported in populations not infected with HIV. The detection of HPV was independent from the time of HIV diagnosis but was positively associated with low CD4 levels, which is in agreement with the correlation between HPV persistence and the host immune status. Such a correlation should cause physicians to be particularly cautious regarding treatment compliance and when considering a modification of ART regimen, whatever the reason. Finally, the high prevalence of infections with various types of HR-HPV in HIV-infected women and the associated risks for cancer support a close cervical screening programme coupled with HPV testing. Regarding vaccination-based prevention, the newly approved nonavalent vaccine including HPV-52 should be preferred.

## Supporting information

S1 Dataset HP2V study’s underlying data set. (XLSX)
